# Methylation of histone H3 lysine 9 occurs during translation

**DOI:** 10.1093/nar/gkv929

**Published:** 2015-09-24

**Authors:** Carlos Rivera, Francisco Saavedra, Francisca Alvarez, César Díaz-Celis, Valentina Ugalde, Jianhua Li, Ignasi Forné, Zachary A. Gurard-Levin, Geneviève Almouzni, Axel Imhof, Alejandra Loyola

**Affiliations:** 1 Fundación Ciencia & Vida, 7780272, Santiago, Chile; 2 Munich Center of Integrated Protein Science and Adolf-Butenandt Institute, 80336 Muenchen, Germany; 3 Institut Curie, Centre de Recherche, Paris, F-75248, France; 4 CNRS, UMR3664, Paris, F-75248, France; 5 Equipe Labellisee Ligue contre le Cancer, UMR3664, Paris, F-75248, France; 6 UPMC, UMR3664, Paris, F-75248, France; 7 Sorbonne University, PSL, France

## Abstract

Histone post-translational modifications are key contributors to chromatin structure and function, and participate in the maintenance of genome stability. Understanding the establishment and maintenance of these marks, along with their misregulation in pathologies is thus a major focus in the field. While we have learned a great deal about the enzymes regulating histone modifications on nucleosomal histones, much less is known about the mechanisms establishing modifications on soluble newly synthesized histones. This includes methylation of lysine 9 on histone H3 (H3K9), a mark that primes the formation of heterochromatin, a critical chromatin landmark for genome stability. Here, we report that H3K9 mono- and dimethylation is imposed during translation by the methyltransferase SetDB1. We discuss the importance of these results in the context of heterochromatin establishment and maintenance and new therapeutic opportunities in pathologies where heterochromatin is perturbed.

## INTRODUCTION

Prior to their incorporation into chromatin, newly synthesized histones proceed through a maturation cascade of several biochemical complexes (1,2). This cascade, mediated by the association with various histone chaperones (3), ensures proper folding and the establishment of post-translational modifications (4). Our previous work, along with others, isolated distinct biochemical complexes, identifying key players that participate in histone maturation (1,2). In the earliest isolated complex, histones H3 and H4 associate with the heat shock proteins Hsc70 and Hsp90/70, respectively, either during their synthesis or once translated. Subsequently, histones H3 and H4 form a heterodimer assisted by the Hsp90 protein and the histone chaperone testicular nuclear autoantigenic sperm protein (tNASP). The dimer then interacts with the histone chaperones anti-silencing function 1 (ASF1) and somatic NASP (sNASP), as well as the histone acetyltransferase 1 (HAT1), consistent with the observation of acetylation at H4K5 and H4K12, a prominent mark of newly synthesized histones (5,6). The H3-H4 dimer then interacts with Importin4, which mediates its translocation to the nucleus (1,2). Importantly, we observed H3K9me1 in each complex, suggesting that this mark is established even earlier in the maturation cascade.

H3K9me1 is a key mark in the establishment of functional heterochromatin (7). Indeed, H3K9me1 is a preferred substrate for the suppressor of variegation 3–9 (SUV39) methyltransferase (8), which catalyzes H3K9me3, a hallmark of heterochromatin and gene silencing (9). Thus, the cell must ensure that certain population of histones feature H3K9me1, priming them for further modifications that enable heterochromatin formation. Consistent with this, SetDB1, a methyltransferase implicated in catalyzing H3K9me1 on soluble histones, exists in a nuclear complex with the histone chaperone chromatin assembly factor 1 (CAF-1) and heterochromatin protein 1 alpha (HP1α) (8). This complex participates in the formation of heterochromatin by (i) depositing the replicative histone variant H3.1-H4 onto newly synthesized DNA, a reaction mediated by CAF-1, and (ii) targeting HP1α to heterochromatin sites (10). Thus, the SetDB1/HP1α/CAF-1 complex links the establishment of histone H3 methylation and heterochromatin formation. Although the SetDB1/HP1α/CAF-1 complex may catalyze H3K9 methylation on soluble histones in the nucleus, it does not explain why we observe H3K9me1 in the first complex isolated in the maturation cascade (1,2). In this work we aimed to identify when H3K9me1 first occurs and the enzyme responsible for its catalysis.

Here we report that H3K9 is mono- and dimethylated while bound to the ribosome and its catalysis occurs co-translationally. We then identified that the methyltransferase SetDB1 associates with ribosomes and catalyzes H3K9me1 and H3K9me2 during translation. Our data enable us to refine our current model, where SetDB1 exists in two distinct complexes, each responsible for catalyzing H3K9me1 at different stages in histone metabolism. We discuss how these two mechanisms cooperate to ensure that a sufficient amount of histones feature H3K9me1 to enable the establishment and maintenance of functional heterochromatin domains. Finally, we discuss how these data open new avenues to explore SetDB1 as a therapeutic target in cancers having perturbed heterochromatin regions.

## MATERIALS AND METHODS

### Antibodies

ASF1a/b (11), CAF-1/p150 (Novus Biologicals #NB500–207A1), DAXX (Santa Cruz #sc-7152), HAT1 (Abcam #ab12164), HIRA (Abcam #ab20655), Histone H3 (Abcam #ab7834), H3K9me1 (Millipore #07–450), H3K9me2 (Millipore, #07–212), H4K12ac (Millipore #07–595), Hsc70 (Abcam #ab19136), Hsp90 (Santa Cruz #sc-7947), Importin4 (Abcam #ab28387), MCM2 (BD Transduction Lab #610700), MCM5 (Bethyl A300–195A), NASP (donated by Dr Almouzni), RPL5 (Abcam #ab74744), RPS3a (Abcam, ab171742), SetDB1 (Abcam #ab12317). For Western blot analysis the primary antibodies were detected with a horseradish peroxidase-conjugated secondary antibody, developed with enhanced chemiluminescence (ECL, Pierce) and exposed onto an X-ray film.

### Ribosome purification

Ribosomes were purified from HeLa cells as described (12). In brief, HeLa cells cultured at 80% confluence were incubated with 0.1 mg/ml cycloheximide during 1 h. After this, cells were trypsinized, PBS washed and lysed with the ribosome lysis buffer (100 mM Tris-HCl pH 7.4, 50 mM KCl, 25 mM MgCl_2_, 0.1 mg/ml cycloheximide, 1 mM DTT, 1 mM PMSF, 1% Triton X-100) during 15 min on ice, and centrifuged at 13 800 g for 15 min. The supernatant was loaded onto a 35% sucrose cushion (10 mM Tris-HCl pH 7.4, 85 mM KCl, 5 mM MgCl_2_, 35% sucrose) and ultracentrifuged in the SW55 rotor (Beckman) for 16 h at 116 000 g, 4°C. The ribosome-containing pellet was resuspended in the ribosome resuspension buffer (10 mM Tris-HCl pH 7.4, 10 mM NaCl, 3 mM MgCl_2_, 0.2 mM DTT).

### Histone processing and mass spectrometry analysis

Histone bands were separated by SDS-PAGE, stained with Coomassie (Brillant blue G-250) and excised at appropriate height. Bands were destained in 50% acetonitrile/50% 100 mM ammonium bicarbonate. Histones were chemically modified by propionylation (30 min, room temperature, 2.5% propionic anhydride (Sigma) in ammonium bicarbonate at pH 7.5) to prevent tryptic cleavage. Histone proteins were then digested with trypsin (Promega, 200 ng in 50 mM ammonium bicarbonate) overnight and the supernatant desalted by Carbon TopTips (Glygen) according to manufacturer's instructions. The peptides were injected in an Ultimate 3000 HPLC system (LC Packings Dionex) and separated with a gradient from 5% to 60% acetonitrile in 0.1% formic acid over 40 min at 300 nl/min on a 75 μm ID X 10 cm ReproSil-Pur C1-AQ analytical column (2.4 μm; Dr. Maisch GmbH Germany). The effluent from the HPLC was directly electro-sprayed into the LTQ Orbitrap XL mass spectrometer (Thermo Fisher Scientific). The MS instrument was operated in the data-dependent mode to automatically switch between full scan MS and MS/MS acquisition. Survey full scan spectra (m/z 250–2000) were acquired in the Orbitrap with resolution R = 60 000 at m/z 400. For all measurements with the Orbitrap detector, three lock-mass ions from ambient air (m/z = 371.10123, 445.12002, 519.13882) were used for internal calibration. The six most intense peptide ions with charge state between two and five were sequentially isolated (window = 2.0 m/z) to a target value of 10 000 and fragmented in the linear ion trap by collision-induced dissociation (CID). Fragment ion spectra were recorded in the linear trap of the instrument. A dynamic exclusion time of 180 s was applied. Typical mass spectrometric conditions were: spray voltage 1.4 kV; no sheath and auxiliary gas flow; heated capillary temperature 200°C; normalized collision energy 35% for CID in linear ion trap. An activation q = 0.25 and activation time of 30 ms were used. Data analysis was performed with XCalibur Qual Browser software (Thermo Fisher Scientific) by using doubly and triply charged peptide masses for extracted ion chromatograms (XICs). XICs were checked manually and values exported to Excel for further calculations as described before (13).

### Histone peptide methyltransferase assay

Recombinant SetDB1 or ribosomes (100 ng/μl RNA) were incubated with 3 μM of histone H3 peptide containing the first 20 amino acids, either unmodified, mono-, di- or trimethylated at K9, in the presence of 60 μM S-adenosyl-methionine (SAM) as the donor methyl group, for 240 min (or as indicated) at 37°C. The reaction buffer contained 25 mM Tris-HCl pH 8.5, 4 mM DTT and 5 mM MgCl_2_. The reaction was stopped by adding tri-fluoroacetic acid to a final concentration of 0.1% (v/v). Methylated peptides were detected by MALDI-TOF mass spectrometry using α-cyano-4-hydroxy-cinnamic acid as the analyte matrix. Its relative abundance was determined using the free-access software *mMass v5.5.0* (14), and expressed as the percentage of the total peptide peak areas. We used the following equation: (AUC H3K9me1 + AUC H3K9me2 + AUC H3K9me3) / (AUC H3 + AUC H3K9me1 + AUC H3K9me2 + AUC H3K9me3), where AUC stands for Area Under the Curve. This number was normalized by subtracting the same percentage determined in the respective non-enzymatic control. Pre-treatment of ribosomes were performed sequentially as followed: incubation for 15 min at 80°C, with 50 mM EDTA for 15 min at 37°C.

### *In vitro* labeling of translating nascent polypeptides

We labeled translating nascent polypeptides as described (15). In brief, ribosomes (4 μg of ribosomal RNA) were incubated with or without 2 μM of biotin-conjugated puromycin (Jena Bioscience) in a buffer containing 10 mM Tris (pH 7,4), 400 mM KCl and 3 mM MgCl_2._ After 90 min at 37°C the reaction was stopped by adding Laemmli Buffer. For treatments, ribosomes were pre-incubated with either 0.1 μg/μl RNAse A or 50 mM EDTA for 15 min at 30°C. The reaction products were analyzed by Western blot using streptavidin-conjugated HRP (Abcam). For pull-down assays, after labeling, the reaction was dialyzed against a buffer containing 10 mM Tris (pH 7,4), 300 mM KCl and 3 mM MgCl_2._ Then, streptavidin conjugated agarose beads (Thermo Scientific) were added, incubated for 90 min and washed with the dialysis buffer containing 0.01% NP40. The reaction products were analyzed by Western blot.

### siRNA treatment

HeLa cells cultured at 80% confluence were transfected with 30 nM of human SetDB1 siRNA duplex (SilencersPre-designed, Ambion, Austin, TX, USA; ID: 138242, sequence: GGGCAGUGACUAAUUGUGAtt) using Lipofectamine 2000 (Invitrogen), according to the manufacturer's instructions. As a negative control, we used 30 nM of negative control siRNA (Silencer Negative Control #1 siRNA, Ambion), a sequence designed without any significant similarity with mouse, rat or human transcripts sequences. 72 h post-transfection, cells were incubated with 0.1 mg/ml cycloheximide during 1 h and ribosomes were purified as described (16). In brief, cells were lysed with the S3 buffer (50 mM Hepes-KOH pH 7.4, 1 mM magnesium acetate, 80 mM potassium acetate, 0.5% Triton X-100, 1 mM PMSF) during 15 min on ice, and centrifuged at 13 800 g for 15 min. The supernatant was loaded onto a 15%–55% sucrose gradient in S3 buffer and ultracentrifuged in the SW41 rotor (Beckman) for 4.5 h at 77 000 g, 4°C. The gradient was then fractioned in 300 μl and each fraction analyzed by 254 nm absorbance, Western blot for RPL5, and agarose gel to detect the 28S and 18S rRNAs.

### Filter H3 peptide methyltransferase assay

1 ug RNA of ribosomes derived from HeLa cells treated with either control or SetDB1 siRNA were incubated with 1 ug of histone H3 peptide containing the first 20 amino acids, in the presence of ^3^H-SAM for 20 min at 30°C. The reaction was stopped with acetic acid, spotted onto P81 filter paper (Whatman), and washed with 50 mM Na_2_CO_3_. The retained cpm was measured using a scintillation counter.

### Immunoprecipitation assay

50 of polysomes RNA were incubated with either 1 μg of antibodies anti-SetDB1, anti-RPS3a or rabbit-IgG as negative control, in a buffer containing 10 mM Tris pH 7.4, 100 mM KCl, 3 mM MgCl_2_ and 0.01% NP40. After incubating with protein A conjugated agarose beads, the beads were washed five times with the incubation buffer. Western blot analyses were performed with the immunoprecipitated samples.

### Expression and purification of recombinant SetDB1

Flag-tagged recombinant human SetDB1 protein was expressed from baculovirus in SF9 cells and purified 72 h post-infection by standard M2 affinity purification. After purification the protein was eluted with 0.2 mg/ml of the Flag peptide and dialyzed against a buffer containing 20 mM Tris-HCl pH 7.9, 10% glycerol, 50 mM KCl, 0.2 mM EDTA, 10 mM B-mercaptoethanol, 0.2 mM PMSF.

## RESULTS

### Histone H3K9 mono- and dimethylation occurs at the ribosome

**Figure 1.**
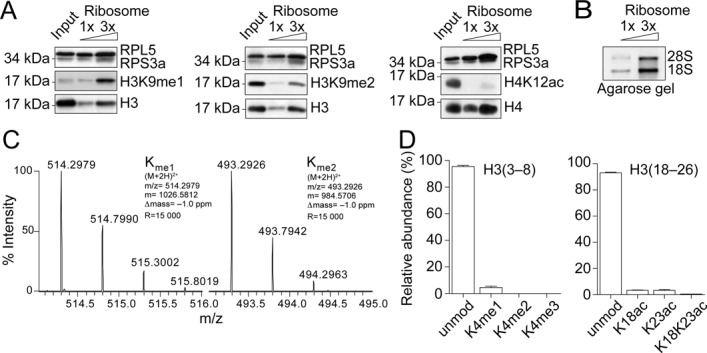
Histone H3K9 mono- and dimethylation occurs at ribosomes. (**A**) Increasing amounts of ribosomes (0.2 and 0.6 ug of ribosomal RNA) were analyzed by Western blot, as indicated. Input corresponded to 0.5% of the total amount of HeLa derived extract loaded onto the sucrose cushion for the purification of ribosomes, corresponding to 15 ug of ribosomal RNA. (**B**) Increasing amounts of ribosomes (0.5 and 1.5 ug of ribosomal RNA) were analyzed by agarose gel for the presence of the 28S and 18S ribosomal RNAs. (**C**) Isotopic distribution of the mono- and dimethylated H3 (9–17) peptides (ion charge = 2+), from which the MS2 spectrum was acquired to locate the methyl groups on K9 (Supplementary Figure S2) (m/z = the mass-to-charge ratio; m = the monoisotopic mass value; Δmass = difference between the excepted and the measured masses; R = resolution of the MS measurement). (**D**) Quantification of the modifications on H3 peptides (3–8) and (18–26) from ribosomal bound histones H3. Standard deviation derived from three independent experiments.

To investigate the timing of H3K9 methylation on newly synthesized histone H3 polypeptides, we isolated and characterized ribosome complexes translating histone H3. This enables us to examine the earliest time point to assess methylation. We enriched for elongating ribosomes by preparing cell extracts from cycloheximide treated HeLa cells and centrifuged them through a 35% sucrose cushion. Western blot analysis of purified ribosomes reveal the presence of the large and small ribosomal subunits RPL5 and RPS3a proteins (Figure 1A), but not β-actin, a cytosolic protein that does not associate with ribosomes (Supplementary Figure S1A). Further characterization reveals 28S and 18S rRNAs on an agarose gel (Figure 1B). Importantly, we confirm the presence of H3 in this ribosome complex (Figure 1A). Further supporting the specific interaction of H3 with mono-ribosomes, we find ribosomes co-eluting with a second peak of histone H3, when centrifuged through a 15–55% sucrose gradient (Supplementary Figure S2, fraction 18). We then used Western blot analysis to probe for the presence of proteins that associate with soluble H3 complexes (1,2). Consistent with previous results (17), we detect the protein Hsc70 (Supplementary Figure S1A), a heat shock protein that binds to nascent polypeptides to facilitate their correct folding. Importantly, we do not detect the histone chaperones ASF1a/b and NASP, the cytoplasmic-nuclear translocator protein Importin-4, nor the minichromosome maintenance (MCM) proteins MCM2 and MCM5 (Supplementary Figure S1A). We also fail to detect the dedicated H3.1 chaperone CAF-1 (18), and two H3.3 chaperones, Hir-related protein A (HIRA) and death associated protein alpha (DAXX) (Supplementary Figure S1B) (3,18–20). Taken together, our data suggest that the isolated ribosome complexes are not contaminated with other soluble chromatin assembly complexes and the associated H3 molecules most likely represent its recent translation.

We then investigated the methylation state of ribosome bound H3 molecules. Interestingly, we detect significant monomethylation of H3K9 (Figure 1A, left), but also a fraction of dimethylated H3K9 (Figure 1A, middle). Though we detect histone H4 associated with ribosomes, we do not observe H4K12 acetylation (Figure 1A, right), a hallmark of newly synthesized histones, consistent with previous work showing that this mark occurs later in the histone processing cascade (1,2). We then exploited mass spectrometry approaches to assess other modifications. Importantly, our analysis confirms that ribosome bound H3 features H3K9me1 and H3K9me2 (Figure 1C and Supplementary Figure S3). Importantly, we do not detect methylation on H3K4 or acetylation on H3K18 or H3K23 (Figure 1D), marks associated with nucleosomal histones. The results support that ribosome-associated histones represent a population of newly synthesized histones in early states of the maturation cascade, rather than parental histones evicted from nucleosomes. Taken together, our data demonstrate that H3K9 methylation occurs while H3 is associated to ribosomes.

### Ribosomes feature H3K9 mono- and dimethyltransferase activity

**Figure 2.**
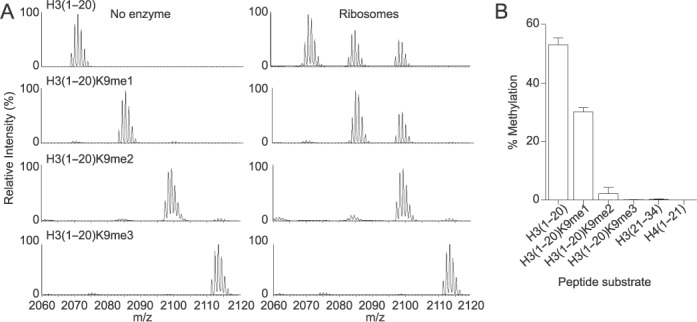
Ribosomes feature H3K9 mono- and dimethyltransferase activity. (**A**) Histone methyltransferase assay using an aliquot of 1 ug (RNA content) of ribosomes (right, labeled as ‘Ribosomes’) or buffer (left, labeled as ‘No enzyme’) mixed with 60 μM SAM and 5 μM of histone H3 peptides from amino acids 1–20 that were unmethylated (H3(1–20)), monomethylated on the residue K9 (H3(1–20)K9me1), dimethylated on the residue K9 (H3(1–20)K9me2), or trimethylated on the residue K9 (H3(1–20)K9me3), for 4 h at 37°C. The reaction was stopped with 0.1% TFA and the methylated products detected by MALDI-TOF mass spectrometry. (**B**) Quantitation analysis of all the methylated peptides for each peptide substrate, expressed as percent of the total H3 peptides, as explained in Materials and Methods. Standard deviation was obtained from three independent assays.

We then asked whether our purified ribosome complexes featured H3K9 methyltransferase activity. To address this question we incubated the purified ribosome complexes with H3 peptide substrates (aa 1–20), where H3K9 was unmodified, mono-, di- or trimethylated, and analyzed the methylation products by MALDI-TOF mass spectrometry. We observe two product peaks shifted by 14 and 28 atomic mass units (amu) with the unmodified substrate, consistent with the addition of one and two methyl groups, respectively, and one product peak shifted by 14 amu with the H3K9me1 substrate. With the di- and trimethylated substrates, we do not observe any product peaks (Figure 2A). We then quantified the extent of methylation under our reaction conditions and calculate 53% methylation of the unmodified H3 peptide (including mono and dimethylation) and 30% methylation of the H3K9me1 peptide (Figure 2B). Notably, when incubating the ribosome complexes with the histone peptides H3 (aa 21–34) and H4 (aa 1–21), we do not observe any product peaks (Figure 2B and Supplementary Figure S4). Together, these data demonstrate that ribosome complexes feature a selective H3K9 mono- and dimethyltransferase activity.

### Co-translational histone H3K9 methylation

**Figure 3.**
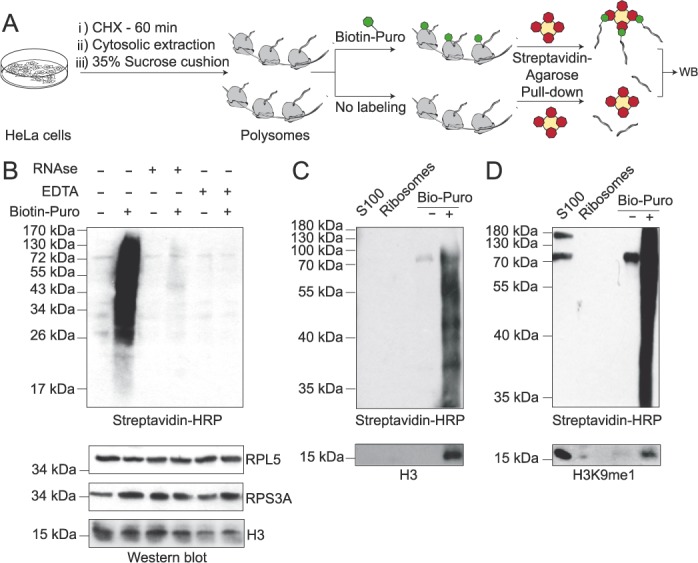
Co-translational histone H3K9 methylation. (**A**) Scheme illustrating the procedure employed to *in vitro* puromycilate nascent polypeptides. (**B**) Western blot analysis of the puromycilation reaction utilizing purified ribosomes, under the conditions described. (**C**–**D**) Western blot analysis of purified biotin-conjugated puromycilated polypeptides, as indicated.

We then assessed whether H3K9 methylation occurs during H3 translation. We isolated elongating ribosomes from cycloheximide treated HeLa cells and *in vitro* labeled translating polypeptides with Biotin-conjugated puromycin (Figure 3A), as previously described (15). We confirmed by Western blot analysis that we only labeled newly synthesized polypeptides upon addition of biotin-conjugated puromycin (Figure 3B, lane 2). Further, when ribosomes were pre-treated with either RNase or EDTA, nascent polypeptides were not puromycilated (Figure 3B, lanes 4 and 6), confirming that active ribosomes are required to label nascent polypeptides. We then purified biotin-puromycin labeled nascent polypeptides with streptavidin-coated beads and probed for H3 polypeptides. We reveal H3, indicating that at least a population of the ribosomal bound histone H3 is in the process of translation (Figure 3C). Importantly, we also detect H3K9me1 on purified nascent polypeptides (Figure 3D). Taken together, we conclude that methylation of H3K9 can occur co-translationally.

### Ribosomes have a SetDB1-dependent H3K9 methyltransferase activity

**Figure 4.**
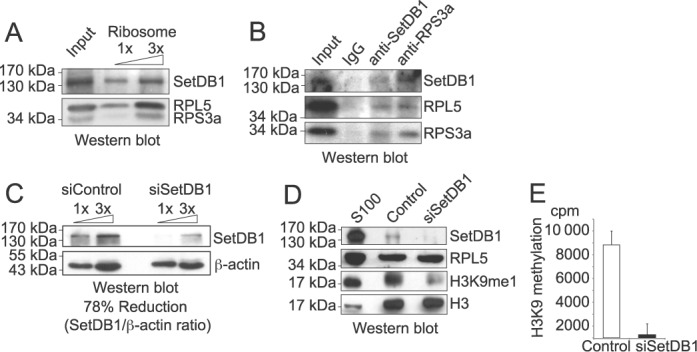
Ribosomes have a SetDB1-dependent H3K9 methyltransferase activity. (**A**) Increasing amounts of ribosomes (0.2 and 0.6 ug of RNA) were analyzed by Western blot, as indicated. (**B**) Western blot analysis of samples derived from immunoprecipitation assays using 50 ug of ribosomal RNA with antibodies against SetDB1 and RPS3a, as well as rabbit antibodies, as negative control. (**C**) Western blot of HeLa cell extracts treated with 30 nM of either control or SetDB1 siRNA for 72 h, blotted as indicated. (**D**) Western blot of ribosomes purified by sucrose gradient from HeLa cells treated with 30 nM of either control or SetDB1 siRNA for 72 h, blotted as indicated. 50 ug of S100 cytosolic extract was loaded as a positive control. (**E**) Histone methyltransferase assay using ^3^H-SAM, 5 μM of histone H3 peptides from amino acids 1–20 that were either unmodified or trimethylated at the lysine 9, and ribosomes purified by sucrose gradient from HeLa cells treated with 30 nM of either control or SetDB1 siRNA for 72 h. H3K9 methylation value was obtained subtracting the cpms obtained in the reaction using H3(1–20)K9me3 peptide from the cpms obtained in the reaction containing the H3(1–20) peptide. Standard deviation was obtained from triplicates.

We previously showed that the methyltransferase SetDB1 catalyzes the monomethylation of H3K9 on soluble histones (8), thus SetDB1 is an attractive candidate to methylate H3 associated to ribosomes. We first demonstrate that SetDB1 associates to the ribosome complex using Western blot analysis (Figure 4A) and co-immunoprecipitation experiments (Figure 4B). We then asked whether this complex featured SetDB1 catalytic activity. To address this, we isolated ribosomes from HeLa cells treated with either a control siRNA or siRNA targeting SetDB1 (siSetDB1). We observe a 78% decrease in SetDB1 upon siRNA treatment compared to control (Figure 4C). Importantly, siRNA treatment does not affect the integrity of ribosomes, as shown by the presence of the ribosomal protein RPL5 (Figure 4D). We then observe by Western blot analysis a decrease in H3K9me1 upon depletion of SetDB1 from ribosomes (Figure 4D). The residual H3K9me1 is likely due to remaining SetDB1. We then assessed the ability of isolated ribosomes depleted of SetDB1 to methylate H3 peptides. Using a radioactive methyltransferase assay, we observed a drastic decrease in methylation upon SetDB1 depletion (Figure 4E). Together, the data suggest that SetDB1 associates to ribosomes and methylates H3K9.

### The ribosomal associated SetDB1 has a non-processive methylation mechanism

**Figure 5.**
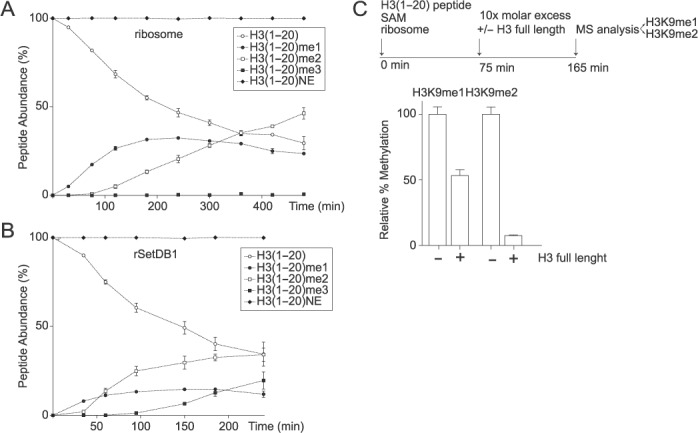
The ribosomal-associated H3K9 HMTase has a non-processive methylation mechanism. Progress curve of histone H3 peptide methylation using unmodified histone H3 peptides from amino acids 1–20 and ribosome complexes (**A**) or recombinant SetDB1 (**B**). Each peptide, unmodified, mono-, di- or trimethylated, was quantified and expressed as percent of the total H3 peptide. NE, non-enzymatic control. Standard deviation was obtained from three independent assays. (**C**) Histone methyltransferase assay using unmodified histone H3 peptides from amino acids 1–20 and ribosome complexes. 75 min after the reaction started, 10X molar excess of histone H3 full length protein was added to the reaction and allowed to proceed for additional 90 min. The methylated peptides (H3K9me1 and H3K9me2) were quantified and expressed as percent of the total H3 peptides. H3K9 HMTase activity of the reaction performed in the presence of competitor was expressed as percent of the reaction performed in the absence of competitor. Standard deviation was obtained from three independent assays.

Given that we observed H3K9me1 and H3K9me2 on ribosomal-bound H3 molecules, we next assessed the mechanism that establishes these marks. Specifically, we asked whether the catalytic activity is processive, where the enzyme remains bound to the substrate during subsequent catalytic turnover (i.e. G9a (21)), or non-processive, where the enzyme dissociates from the substrate after catalytic turnover prior to catalyzing the second methyl transfer (i.e. SUV39 (22)). We used MALDI-TOF MS to examine simultaneously the decrease in the starting unmethylated substrate, the formation of the H3K9me1 intermediate, and the formation of the H3K9me2 product. Under steady-state conditions, if SetDB1 methylates the peptide in a processive manner, we would not detect the H3K9me1 intermediate, and the substrate depletion and product formation curves would reciprocate each other. Conversely, if SetDB1 behaves in a non-processive manner, we should detect the accumulation of the H3K9me1 intermediate, and a lag phase for the formation of the H3K9me2 final product. Indeed, we observe a transient accumulation of the H3K9me1 peptide, supporting the non-processive mechanism (Figure 5A). We observe similar behavior using recombinant SetDB1, which also trimethylates H3K9 (Figure 5B). Finally, we assessed whether the addition of excess full-length H3 molecules after reaction initiation acted as a competitive inhibitor. Indeed, we observe a decrease of the H3K9me2 product with 10-molar excess of full-length histone H3 (Figure 5C), further supporting the non-processive mechanism. Taken together, our data demonstrate that SetDB1 associates to ribosomes to establish the mono- and dimethylation mark on H3K9 during translation in a non-processive manner.

## DISCUSSION

Here, we report that mono- and dimethylation of H3K9 occurs while H3 is associated to ribosomes (Figure 1) and that the reaction is catalyzed during H3 translation (Figure 3) by the SetDB1 methyltransferase (Figure 4). This is, to the best of our knowledge, the first histone modification shown to occur during translation, supporting the critical nature of this mark for genome integrity.

### SetDB1 establishes the H3K9 methylation mark on ribosomes by a non-processive mechanism

We show that ribosomal-associated SetDB1 acts via a non-processive mechanism to catalyze H3K9me1 and H3K9me2 (Figure 5). Interestingly, with the exception of SUV39 (22,23), most H3K9 methyltransferases, including G9a (21) and the neurospora DIM-5 (24), utilize a processive mechanism to methylate their substrates. We hypothesize that the non-processive methylation mechanism enables a level of regulation toward the extent of methylation on ribosomes. Further work should investigate whether the speed of mRNA translation might bias methylation toward either mono or dimethylation. For example, if H3 polypeptide synthesis and/or protein folding is fast, potentially during S-phase, the formation of H3K9me1 would be favored over H3K9me2. Given that recombinant SetDB1 can trimethylate H3K9 (Figure 5), it would also be interesting to consider that under stress conditions trimethylation may be possible if there is a delay in histone translation. This could potentially bypass the need for SUV39 to expedite heterochromatin formation or alternatively may act as a signal for defective histones that need to be degraded.

### Two mechanisms to establish newly synthesized H3K9 methylation

**Figure 6.**
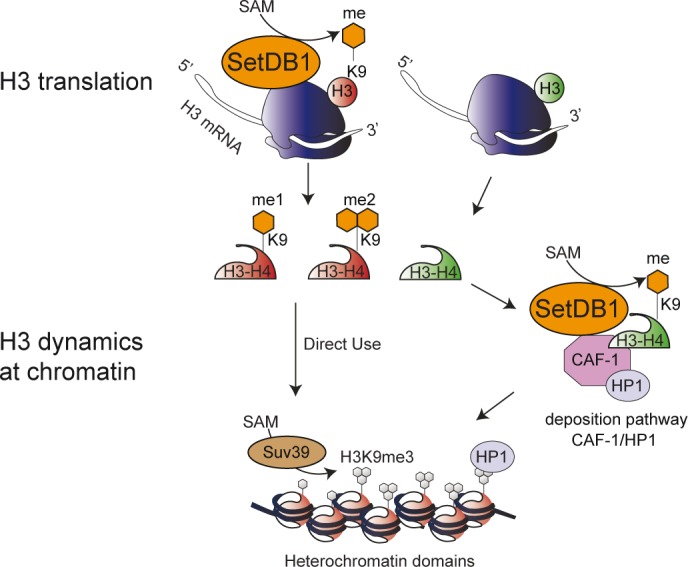
Working model: SetBD1 exists in two distinct complexes to establish H3K9 methylation to potentiate the final chromatin modification state. In one complex, SetDB1 is bound to a population of ribosomes and methylates H3K9 during H3 polypeptide translation. This methylation would ensure that a fraction of H3 features a particular pattern of modifications allowing the subsequent methylation by SUV39 to the H3K9me3 state. We propose that the SetDB1/HP1α/CAF-1 complex, which monomethylates H3K9 during heterochromatin replication, may also act as a contingency mechanism to ensure sufficient H3K9 methylation enabling proper heterochromatin establishment and maintenance.

It is interesting to consider that SetDB1 acts by two distinct mechanisms to methylate H3K9 (Figure 6). The first mechanism, reported here, establishes H3K9me1/2 during translation. This creates an immediate pool of newly synthesized histones that once deposited onto DNA serve as a template for additional modifications that will give rise to heterochromatin domains. Of course, one cannot rule out that newly synthesized H3K9 methylation might play other roles as well, including histone folding, the regulation of protein–protein interactions or histone degradation. Secondly, we previously reported a SetDB1/HP1α/CAF-1 complex that monomethylates soluble H3K9 as a mechanism to ensure heterochromatin maintenance after replication or DNA damage repair (8). This complex may operate through two distinct mechanisms: (i) to handle parental histones evicted from chromatin, using SetDB1 to methylate H3K9, prior to recycling the parental histones onto newly synthesized DNA, and (ii) as a contingency plan in case there are errors in the metabolism of newly synthesized histones. Future work should consider how the balance of SetDB1 in those two complexes shifts through the cell cycle and the resulting functional implications such as heterochromatin integrity. Interestingly, SetDB1 is overexpressed in several cancers including human melanomas (25), and the SetDB1 gene is amplification in some lung tumors (26,27). The change in the dosage of SetDB1 could further impact the balance of the SetDB1 protein in the two complexes and lead to aberrant or perturbed heterochromatin domains. Furthermore, SetDB1 depletion using RNAi approaches in cancer cell lines or mouse xenograft models leads to a decrease in proliferation (27). Therefore, SetDB1 could represent a new therapeutic target in tumors overexpressing SetDB1 or with heterochromatin defects. Taken together, our work characterized for the first time a mechanism to establish histone methylation during translation, and highlights the importance of considering histone dynamics outside of chromatin as critical contributors to genome integrity.

## Supplementary Material

SUPPLEMENTARY DATA
