# Supplementary material for: Methylation of histone H3 lysine 9 occurs during translation
Source: Nucleic Acids Res. 2015 Sep 24;43(19):9097–106. doi: 10.1093/nar/gkv929 (PMC4627087; doi:10.1093/nar/gkv929)
Supplement: SUPPLEMENTARY DATA [file supp_43_19_9097__index.html]

Methylation of histone H3 lysine 9 occurs during translation — SUPPLEMENTARY DATA 

# Methylation of histone H3 lysine 9 occurs during translation

## SUPPLEMENTARY DATA

- SUPPLEMENTARY DATA
