# Supplementary material for: Methylation of histone H3 lysine 9 occurs during translation
Source: Nucleic Acids Res. 2015 Sep 24;43(19):9097–106. doi: 10.1093/nar/gkv929 (PMC4627087; doi:10.1093/nar/gkv929)

SUPPLEMENTAL INFORMATION

SUPPLEMENTAL FIGURES

Supplementary Figure 1. Histone H3 and ribosome interaction is specific. (A) Increasing amounts of ribosomes (0.2 and 0.6 ug of ribosomal RNA) were analyzed by Western blot, as indicated. Input corresponded to 0.5% of the total amount of HeLa derived extract loaded onto the sucrose cushion for the purification of ribosomes, corresponding to 15 ug of ribosomal RNA.

Supplementary Figure 2. Purification of ribosomes. We purified mono-ribosomes from S3 extract derived from cycloheximide treated HeLa cells on a 15%-55% sucrose gradient. After ultracentrifugation, we fractionated the gradient and analyzed the fractions by 254 nm absorbance (top), agarose gel to detect the 28S and 18S rRNAs (middle top), Ponceau red staining of PVDF membrane (middle bottom), and Western blot for RPL5 and histone H3 (bottom).

Supplementary Figure 3. Identification of the methylated H3K9 residue in ribosomal bound histone H3. The MS2 fragmentation spectrum of the mono and dimethylated H3 (9-17) peptide (upper and bottom, respectively). The observed y and b ion series provide high confidence in the localization of the methylation on H3K9.

Supplementary Figure 4. Ribosomes feature H3K9 mono- and dimethyltransferase activity. Histone methyltransferase assay using an aliquot of 1 ug (RNA content) of ribosomes (right, labelled as “Ribosomes”) or buffer (left, labelled as “No enzyme”) mixed with 60 μM SAM and 5 μM of histone H3 peptides from amino acids 1-20 that were unmethylated (H3(1-20)), monomethylated on the residue K9 (H3(1-20)K9me1), dimethylated on the residue K9 (H3(1-20)K9me2), or trimethylated on the residue K9 (H3(1-20)K9me3); from amino acids 21-34, or histone H4 peptide from amino acids 1-21, for 4 h at 37^{0}C. The reaction was stopped with 0.1% of TFA and the methylated products detected by MALDI-TOF mass spectrometry. Quantitation analysis of the methyl groups incorporated into the different peptide substrates is shown in Figure 2D.

Supplementary Figure 1: Histone H3 and ribosome interaction is specific

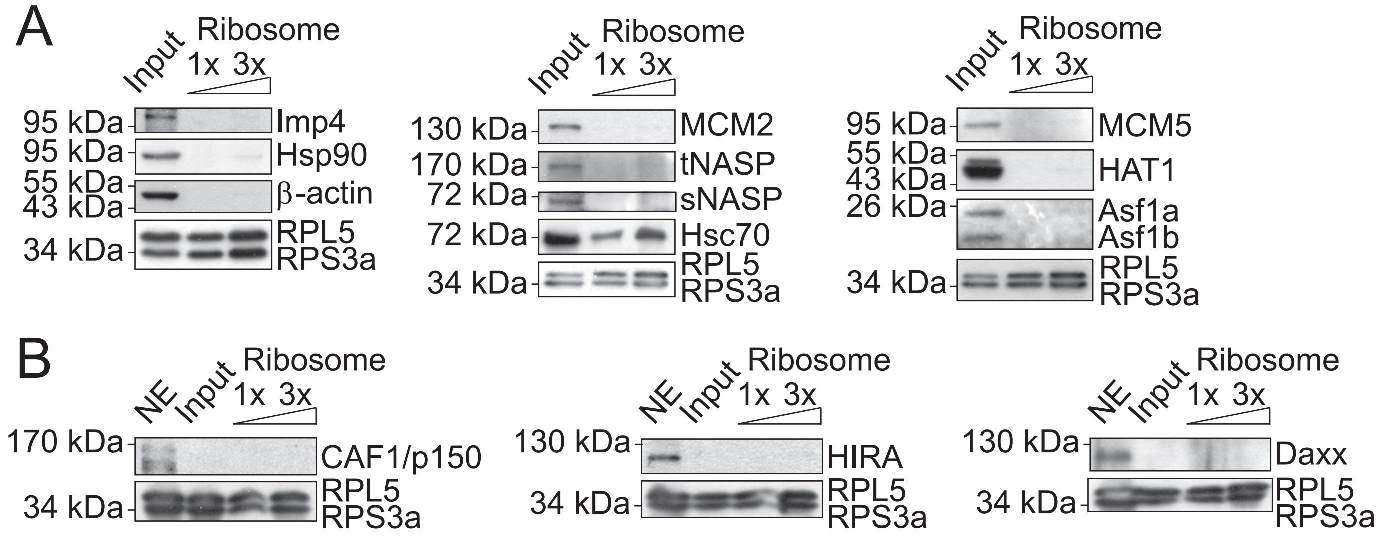

Supplementary Figure 2: Purification of ribosomes

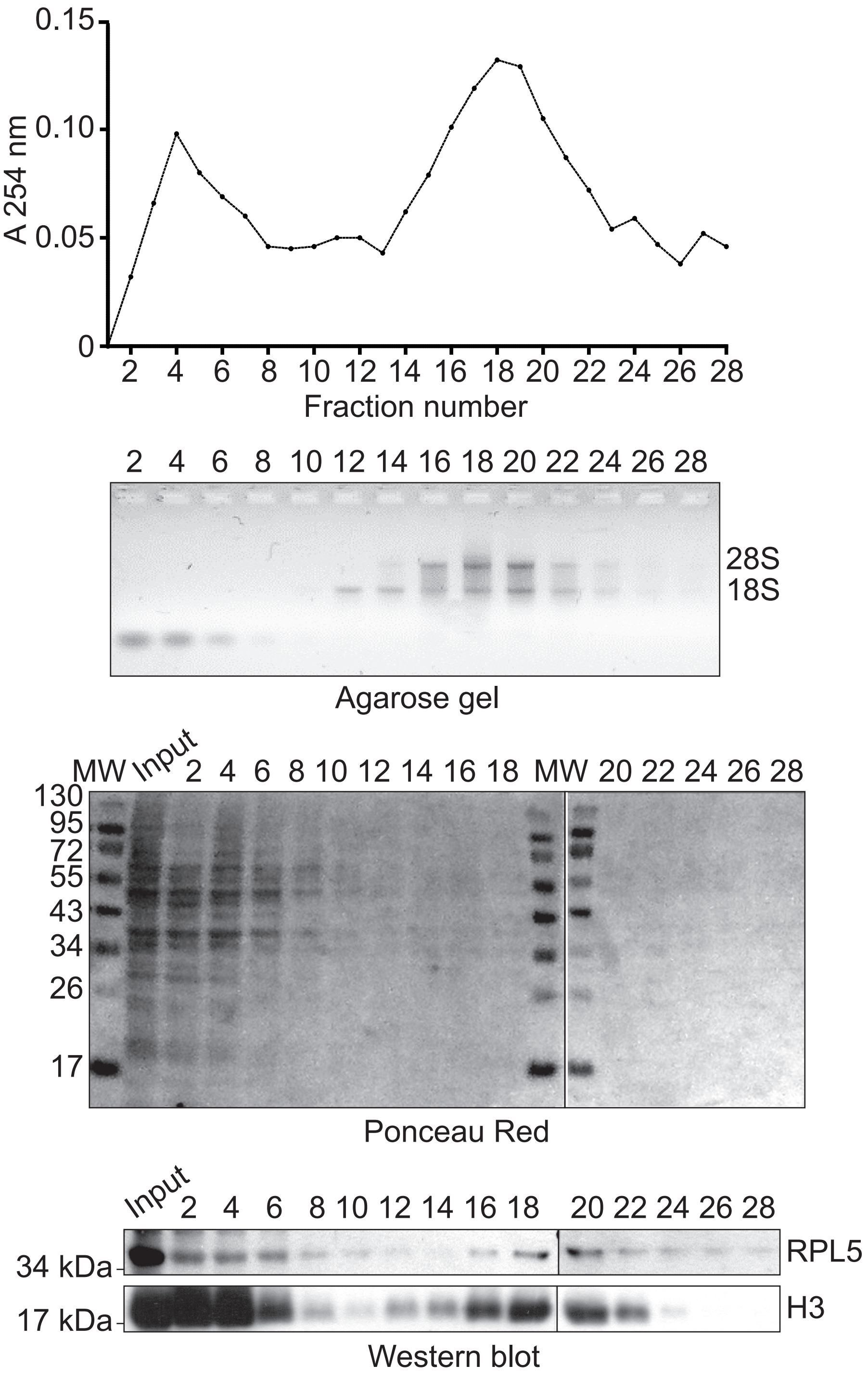

Supplementary Figure 3: Identification of the methylated H3K9 residue in ribosomal bound histone H3

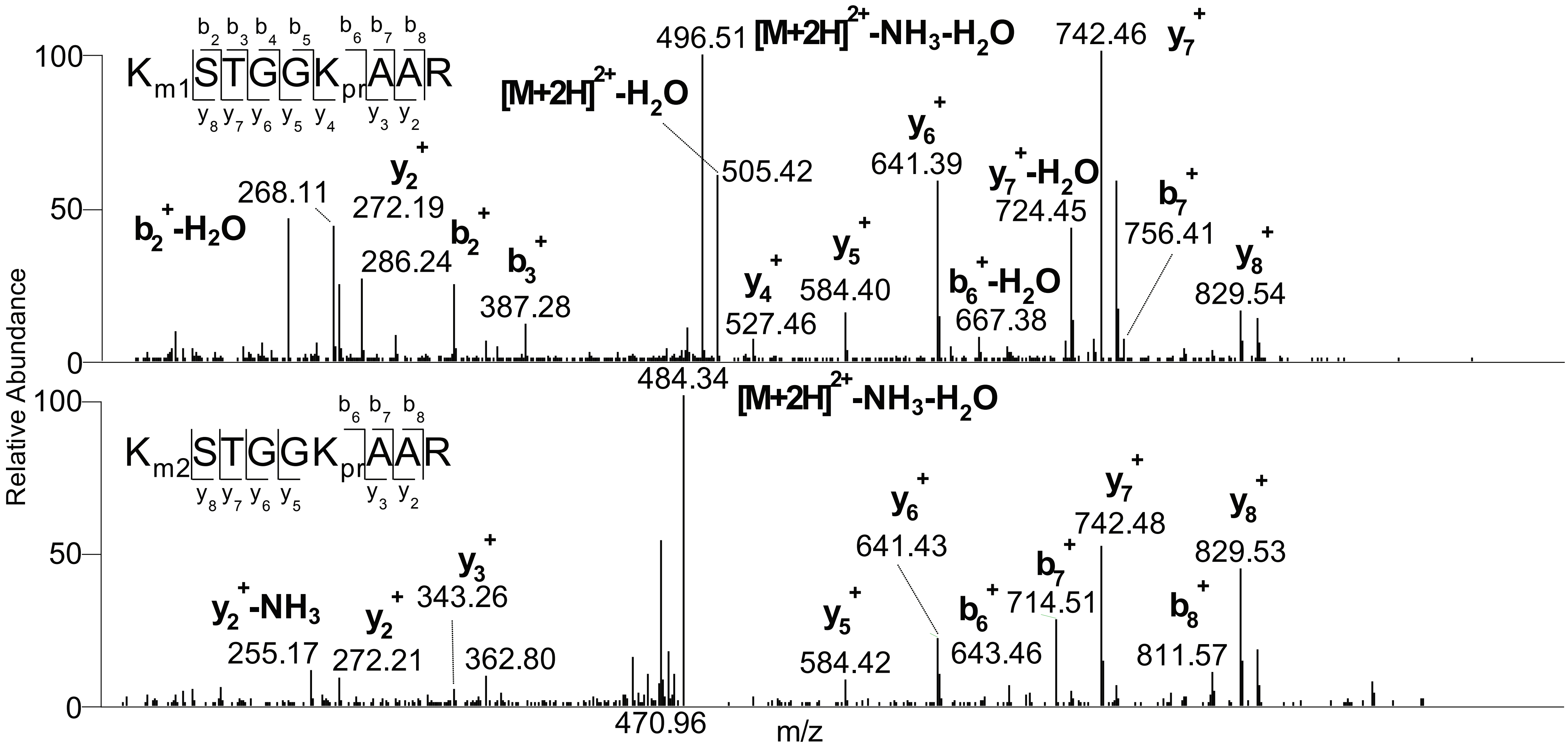

Supplementary Figure 4: Ribosomes feature H3K9 mono- and dimethyltransferase activity

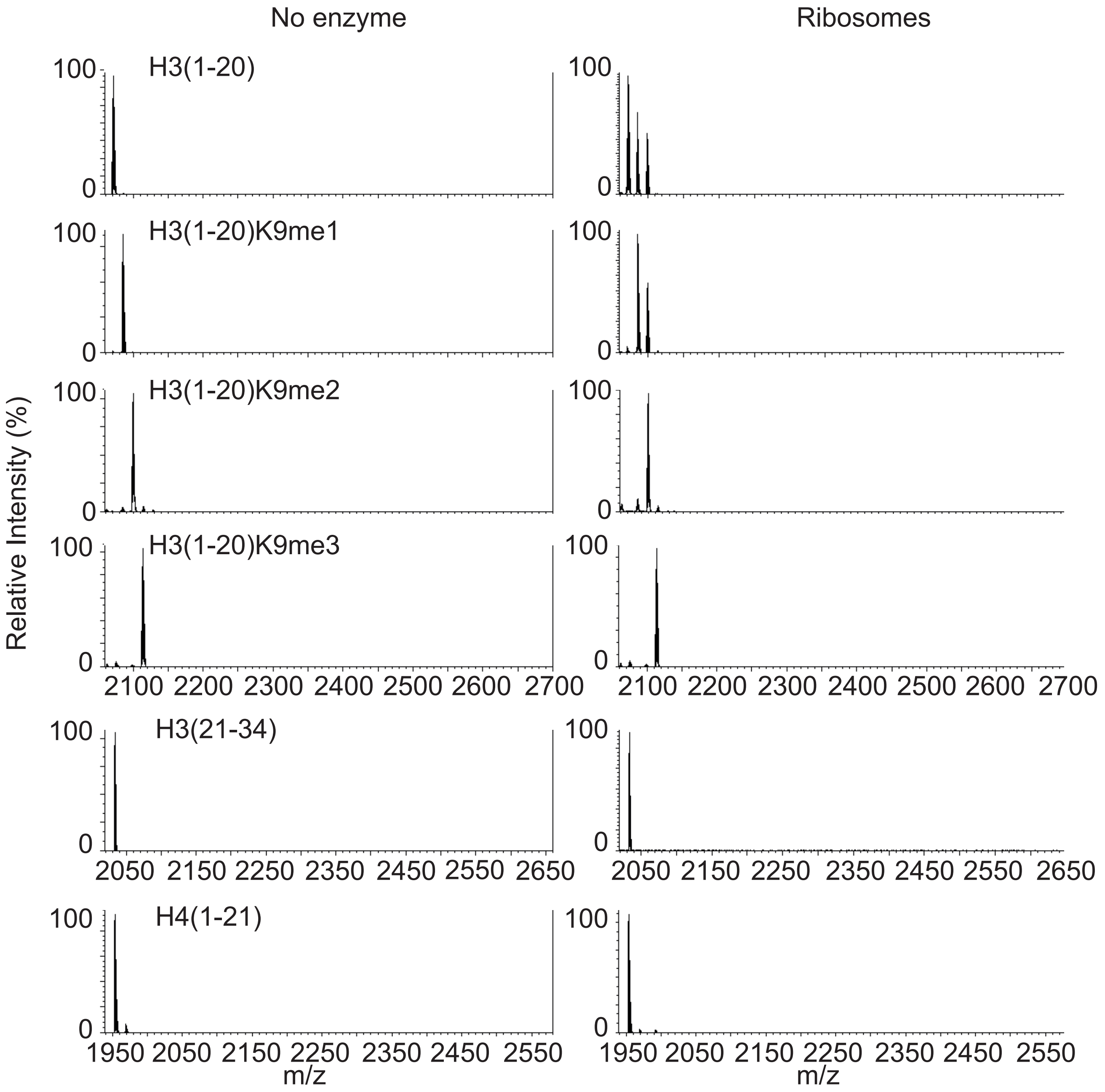

Supplement: SUPPLEMENTARY DATA [file supp_gkv929_nar-00909-x-2015-File008.pdf]
